# Impact of Prescription Opioid Detoxification on Quality of Life and Pain Levels

**DOI:** 10.3390/clinpract14040123

**Published:** 2024-08-08

**Authors:** Gabija Laubner Sakalauskienė, Indrė Stražnickaitė, Sigutė Miškinytė, Linas Zdanavičius, Jūratė Šipylaitė, Robertas Badaras

**Affiliations:** 1 Clinic of Anaesthesiology and Intensive Care, Institute of Clinical Medicine, Faculty of Medicine, Vilnius University, LT-03101 Vilnius, Lithuania; jurate.sipylaite@mf.vu.lt (J.Š.); robertas.badaras@mf.vu.lt (R.B.); 2 Faculty of Medicine, Vilnius University, LT-03101 Vilnius, Lithuania; indre.straznickaite@gmail.com (I.S.); sigute.miskinyte@gmail.com (S.M.); linas.zdanavicius@gmail.com (L.Z.)

**Keywords:** prescription opioids, detoxification, quality of life, chronic pain

## Abstract

Purpose: The aim of this study was to examine the impact of detoxification from prescription opioids on the quality of life (QoL) and pain levels among patients reliant on these medications for chronic pain management. Objective: Long-term use of opioids for pain management may lead to a range of adverse effects, including tolerance, dependence, significant societal costs, and a decline in overall quality of life (QoL). Despite these challenges, there is a limited amount of research focusing on the effects of detoxification and its impact on the QoL for patients with chronic pain tolerant to prescription opioids. Methods: This prospective study included 45 patients who underwent elective detoxification from prescription opioids. Prescription opioids were discontinued during the detoxification treatment in 44 of the 45 cases. QoL was monitored using SF-36v2™ questionnaires administered before detoxification, on the day of discharge, and at least six months after detoxification. Pain levels were assessed using Visual Analogue Scale (VAS) scores before and after the detoxification process. Results: The study was fully completed by 30 patients. At the third SF-36v2™ evaluation, 25 out of 30 patients (83.3%) reported the detoxification treatment as beneficial to their overall health status compared to that before the treatment, and SF-36v2™ questionnaires after detoxification were significantly higher than before the treatment (*p* < 0.001). A decreased pain level right after the detoxification was reported by 44 of the 45 patients (97.7%), with a significant average reduction of 4.51 points observed (*p* < 0.001). Conclusions: The observed enhancement in QoL, significant reduction in pain, and cessation of opioid use in most patients with chronic pain tolerant to prescription opioids following opioid detoxification indicate that this method of treatment can be safely and effectively administered and must be considered for chronic pain patients.

## 1. Introduction

Chronic pain management frequently depends on the prescription of opioids, which, despite their effectiveness in pain alleviation, are associated with a significant range of adverse effects, including tolerance, dependence, substantial societal costs, and a decline in overall quality of life (QoL) [1]. Furthermore, opioids demonstrate limited effectiveness in the long-term management of chronic noncancer pain (CNCP). A multicenter, prospective cohort study observed no improvement in pain symptoms, physical function, emotional function, or social/familial disability among opioid users over a two-year period [2].

Despite the widespread use of opioids and the recognition of their potential for addiction, research examining the impact of detoxification on QoL, and pain management outcomes remains limited. Detoxification, the process of medically supervised withdrawal from opioids, is often viewed with skepticism due to concerns about pain management and the potential for relapse [3]. However, there is a growing interest in understanding whether successful detoxification can lead to improvements in QoL by mitigating the adverse effects of long-term opioid use.

Both physicians and patients often fear starting prescription opioids due to concerns about addiction and the difficulty of stopping these medications once they begin using them. Studies show that the risk of addiction in chronic pain patients varies widely, with some estimates as low as 3–4% and others as high as 8–12% [4,5]. This wide range reflects the complexity of addiction, which is influenced by a combination of physical, psychological, and genetic factors [4,6]. Patients’ concerns are not unfounded. Physical dependence on opioids can lead to withdrawal symptoms if the medication is stopped abruptly, while tolerance can necessitate higher doses over time to achieve the same level of pain relief [7]. These factors contribute to the challenge of ceasing opioid use once dependence has developed [8].

Patients often experience a paradoxical pain state where the use of opioids leads to heightened pain sensitivity, making pain management more challenging and sometimes necessitating higher doses, which can further aggravate the condition [9]. This creates a vicious cycle of pain and opioid dependency, further diminishing the patient’s quality of life. Opioid-induced hyperalgesia (OIH) is a condition where opioid medications, which are intended to relieve pain, paradoxically cause increased sensitivity to pain. This condition can significantly impact the quality of life for patients using prescription opioids. Research indicates that OIH is characterized by a reduction in pain threshold and tolerance, leading to heightened pain sensitivity and higher pain scores [10]. This is observed in both chronic and surgical pain patients treated with opioids like remifentanil, fentanyl, and morphine [11].

A comprehensive review of opioid use for chronic pain indicates that pain levels can increase substantially during attempts to taper off opioids, which can drive patients to increase their dosage to alleviate the pain [12,13]. This phenomenon is supported by studies showing that patients often struggle with inadequate pain control during tapering, which can result in higher dropout rates from treatment programs due to both increased pain and the side effects of opioids [12]. Some patients require significantly higher doses to achieve the same level of pain control, and when they attempt to taper, the resulting pain can be severe enough to undermine their motivation to continue the process. This cycle of increasing pain and escalating opioid use can lead to feelings of hopelessness and frustration, making it difficult for patients to maintain their commitment to cessation.

Genetic factors play a significant role in opioid addiction susceptibility [14]. Certain genetic variations can influence how individuals metabolize and respond to opioids, affecting their risk of developing addiction [14]. The exact dose and duration of opioid use that consistently lead to addiction remain uncertain; nevertheless, it is clear that the likelihood of opioid addiction significantly differs among individuals, with genetic susceptibility contributing to at least 35 to 40% of the overall risk [15,16]. Understanding these genetic factors can help in developing personalized approaches to pain management and addiction treatment [17].

Detoxification treatments, when managed, can potentially improve QoL by reducing dependence on opioids and minimizing their negative health impacts [18]. However, the process is complex and requires comprehensive support, including pain management alternatives and psychological support to address both physical and emotional aspects of dependency [18]. The growing focus on wellness underscores the importance of measuring quality of life in addition to substance use outcomes [19].

In summary, the cycle of increased pain during opioid withdrawal either tapering, coupled with the need for higher doses to manage that pain, can significantly reduce patients’ motivation to cease opioid use, leading to a cycle of increased dependence and diminished motivation to quit. Understanding these risks and the measures to mitigate them can help patients and healthcare providers make more informed decisions regarding pain management and the use of prescription opioids. In the context of detoxifying opioid-dependent chronic pain patients, it is crucial to adopt short-term detoxification strategies that do not exacerbate pain intensity throughout the process.

## 2. Materials and Methods

This prospective study, conducted from 2019 to 2023, recruited a cohort of 45 patients undergoing prescription opioid detoxification at the Toxicology Centre of Republican Vilnius University Hospital, Lithuania. All 45 patients completed the study. The study protocol was approved by the Vilnius Regional Committee on Biomedical Research Ethics (license no. 2019/10-1153-644), and written informed consent was obtained from all patients before inclusion. Referrals to the Toxicology Centre originated from primary care physicians and secondary care facilities, including the National Centre for Cancer and Pain clinics, with patients subsequently assessed for suitability for opioid detoxification.

Patients who met the following criteria were included in the study: a documented opioid tolerance and established dependence to prescription opioids and admission to the Toxicology Centre for elective opioid detoxification. Opioid tolerance was referred to as a diminished analgesic response over time, necessitating higher doses to achieve the same level of pain relief. Conversely, patients were excluded from the study based on the following criteria: acute opioid poisoning, diagnosed addiction to illicit opioids, and diagnosed addiction to multiple psychoactive substances.

Upon admission, pertinent clinical and demographic information was gathered. This information encompassed details such as the patient’s age, the duration of their pain, their primary pain location or diagnosis, their opioid consumption, and the length of time they had been using opioids. The opioid dosage at admission was ascertained through a combination of patient self-report and a review of their medical records. For ease of analysis, the opioid intake was subsequently converted into oral morphine equivalents.

This detoxification protocol was designed with a reference to our earlier work [20], which relied on benzodiazepine (diazepam) and α2 agonist (clonidine) administration at the opioid abstinence expression level that was being assessed and adjusted according to the Objective Opioid Withdrawal Scale and the Subjective Opioid Withdrawal Scale [21]. Opioid medications are discontinued during detoxification treatment, and patients are closely monitored for withdrawal symptoms, with a timely and carefully titrated intervention to manage early withdrawal symptoms. We included nonsteroidal anti-inflammatory drugs (ibuprofen, diclofenac, and ketorolac), antipsychotics (haloperidol and quetiapine), anticonvulsants (gabapentin), and tetracyclic antidepressants (mirtazapine), with the doses titrated for each patient individually according to their opioid withdrawal symptoms, in the treatment and continuous monitoring throughout the detoxification process. Contrary to the referenced original protocol, naltrexone was not used for patient treatment in this study.

QoL was assessed using the SF-36v2™ questionnaire in Lithuanian language [22,23,24] at three distinct time points: before detoxification, on the day of discharge, and at least six months post-detoxification (follow-up telephone call). The QoL SF-36v2™ questionnaire consists of 36 items grouped into eight scales: physical functioning, role limitations due to physical health, role limitations due to emotional problems, energy/fatigue, emotional wellbeing, social functioning, pain, and general health. Each scale is scored from 0 to 100, with higher scores indicating better QoL. The overall QoL score is the average of these eight scales.

Patient pain intensity was measured with the Visual Analogue Score (VAS). VAS used in this study ranged from 0 to 10, where 0 indicated no pain and 10 represented the worst pain imaginable (Table 1). We measured pain only on the day before detoxification and on the day of discharge after detoxification procedure.

The analysis of data was conducted utilizing MS Excel and IBM SPSS 23.0 software. Continuous data are presented as the mean with accompanying standard deviation, while proportions are expressed in percentages. The independent *t*-test was employed for the comparison of means between groups. Statistical significance was considered achieved when *p* < 0.05.

## 3. Results

Out of the 45 patients, 44 successfully completed the detoxification process, with 28 women, constituting 63.63% of the cohort. The average age of the participants was 53.6 ± 12.8 years. The detoxification process itself had an average duration of 9.2 ± 3.2 days. Participants had a history of prescribed opioid usage spanning an average of 57.8 ± 66.9 months (Table 2).

Opioid use was most commonly for headaches (14/44) and cancer (14/44), followed by back pain (10/44). Less frequent reasons included rheumatoid arthritis (2/44), gastrointestinal pathology (2/44), and other conditions: muscle pain (1/45), and humerus injury (1/45) (Table 2).

The data show a diverse range of prescribed opioids, with Tramadol being the most prescribed (12 patients), followed closely by Codeine and Morphine to nine each. Less frequently prescribed opioids included Fentanyl (transdermal) to four, Pethidine to two, and Methadone and Oxycodone to one each. Several patients used multiple opioids: two patients received Morphine and Fentanyl, two received Fentanyl and Tramadol, one received Morphine and Tramadol, and one received Codeine, Morphine, and Tramadol (Table 2).

The prescribed dose of opioids averaged 142 ± 157 mg per day in oral morphine equivalent doses (MEDs). For single opioids, the MEDs ranged from 20.54 mg (±22.71) for Codeine to 450 mg for Oxycodone. For combined opioid therapies, the MEDs varied, with highest being 315 mg (±75) for the combination of Morphine and Fentanyl (Table 3).

### 3.1. Quality of Life Assessment

**Figure 1:**
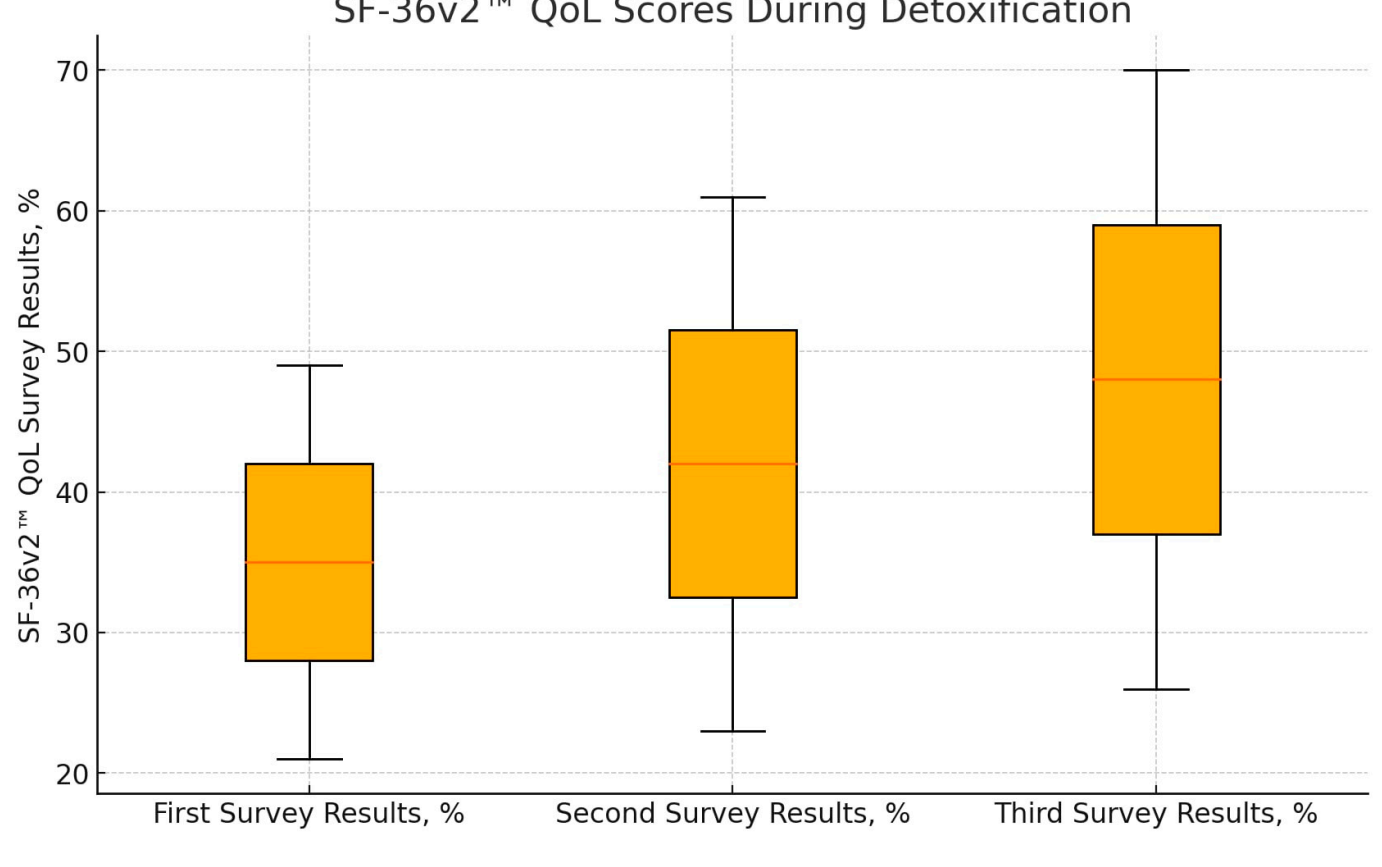
Results of SF-36v2™ QoL questionnaires during detoxification (n = 30). There is a statistically significant difference between the estimates of the first and second questionnaires (*p* = 0.004) and between the first and third questionnaires (*p* < 0.001) and between the second and third questionnaires (*p* = 0.025).

Full follow-up was obtained for 30 patients, with the remaining 15 patients either declining to complete the third SF-36v2™ form or being uncontactable. Statistical analysis revealed a significant improvement in QoL scores across all three time points. The mean QoL scores were as follows: first—before detoxification, 35 ± 14%; second—on the day of discharge, 42 ± 19% (*p* = 0.004 compared to predetox); and third—at least six months after detoxification (follow-up call), 48 ± 22% (*p* < 0.001 compared to predetox; *p* = 0.025 compared to discharge). (Figure 1). These results indicate a continuous improvement in QoL from predetoxification to post-detoxification, demonstrating the beneficial impact of opioid detoxification on patients’ overall wellbeing.

### 3.2. Pain Scores

**Figure 2:**
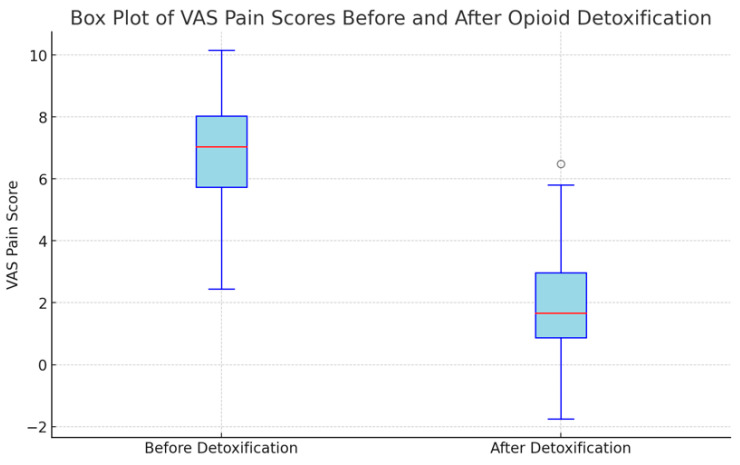
Results of VAS pain score before and after detoxification (n = 44).

The Visual Analog Scale (VAS) pain scores were measured for 44 patients before and after undergoing opioid detoxification. The mean VAS scores were 6.68 ± 2.0 points before detoxification and 2.17 ± 1.93 points after detoxification (*p* < 0.001). This average reduction of 4.51± 1.83 points underscores a substantial decrease in pain levels following the detoxification process (Table 2 and Figure 2).

### 3.3. Opioid Usage Cessation

Among the 45 patients, 44 (97.7%) successfully ceased opioid use following the detoxification program. This high success rate underscores the effectiveness of the detoxification process in eliminating opioid dependence in patients with chronic pain.

## 4. Discussion

The significant improvements in QoL and reductions in pain levels post-detoxification indicate that, despite the challenges associated with opioid withdrawal, successful detoxification can yield substantial positive outcomes [25,26].

The observed improvement in QoL scores across the three assessment points demonstrates the beneficial impact of opioid detoxification. Before detoxification, patients had significantly lower QoL scores, reflecting the detrimental effects of long-term opioid use on their physical and emotional wellbeing [27,28]. The increase in QoL scores on the day of discharge and six months post-detoxification suggests that the benefits of detoxification are not only immediate but also sustained over time. This aligns with findings from previous research, which indicate that reducing opioid dependence can lead to better overall health outcomes and improved patient satisfaction [29].

A significant reduction in pain levels, as measured by the VAS, was observed following detoxification. This finding is particularly noteworthy given the common concern that withdrawal from opioids might exacerbate pain symptoms [26]. The average reduction of 4.51 points on the VAS scale highlights the effectiveness of the detoxification process in managing pain, contradicting the notion that opioid cessation invariably leads to worsened pain outcomes. This reduction in pain could be attributed to the resolution of opioid-induced hyperalgesia (OIH), where long-term opioid use paradoxically increases pain sensitivity. By eliminating the opioids from the system, patients may experience a normalization of their pain thresholds, contributing to the observed pain relief [29].

The high success rate of opioid cessation among the study participants (97.7%) further supports the effectiveness of the detoxification protocol. This outcome is particularly significant given the substantial challenges associated with opioid tolerance and withdrawal. The structured detoxification protocol, which included the use of benzodiazepines, α2 agonists, nonsteroidal anti-inflammatory drugs, antipsychotics, anticonvulsants, and antidepressants, was crucial in managing withdrawal symptoms and facilitating successful opioid cessation. Previous studies have highlighted the importance of comprehensive support during detoxification to address both the physical and psychological aspects of dependency [28].

## 5. Limitations

While the results of this study are promising, several limitations must be acknowledged. The sample size was relatively small, and the study was conducted at a single center, which may limit the generalizability of the findings. Additionally, the follow-up period was limited to six months, and longer-term outcomes were not assessed. Furthermore, the study did not assess whether there was an improvement in VAS pain scores or the necessity for opioids among patients beyond the initial 6-month detoxification period. It is important to note that VAS pain scores measurements were not conducted after this 6-month period, as they could be confounded by additional variables, including the use of other NSAID medications.

## 6. Conclusions

This study provides compelling evidence that elective opioid detoxification can lead to significant improvements in QoL and reductions in pain levels for patients with chronic pain tolerant to prescription opioids. The high success rate of opioid cessation further underscores the potential benefits of a structured detoxification protocol. Despite the challenges associated with opioid withdrawal, the findings suggest that with appropriate support and management, detoxification can be a viable and beneficial option for patients struggling with opioid dependence. These results advocate for broader implementation of detoxification programs to improve the wellbeing and health outcomes of patients tolerant to prescription opioids, while differentiating this population from those with advanced cancer or other conditions where opioid use is clinically necessary.

## Figures and Tables

**Table 1:** VAS scoring criterion.

VAS Score	Pain Description
0	No pain
1–3	Mild pain
4–6	Moderate pain
7–9	Severe pain
10	Worst pain imaginable

**Table 2:** Patient characteristics.

**Average length of opioid usage (months ± SD)** (n = 44)		57.8 ± 66.9
**Average detoxification duration (days ± SD)** (n = 44)		9.2 ± 3.2
**Pain level** (n = 44)		
VAS before detoxification (points ± SD)		6.68 ± 2
VAS after detoxification (points ± SD)		2.17 ± 1.93
**Reason for opioid use; pain-causing disease** (n = 44)		
Oncological disease		14 (31.81%)
Neurological disease		14 (31.81%)
Musculoskeletal disease		10 (22.72%)
Rheumathoidal disease		2 (4.54%)
Gastrointestinal		2 (4.54%)
Other disease		2 (4.54%)
VAS for each disease **before and after detoxification (n = 44)** (points ± SD)		
Oncological disease (n = 14)	5.9 ± 2.59	1.60 ± 1.88
Neurological disease (n = 14)	6.07 ± 1.55	1.78 ± 1.63
Musculoskeletal disease (n = 10)	7.7 ± 2.13	2.6 ± 2.46
Rheumathoidal disease (n = 2)	6.75 ± 1.06	2.5 ± 0
Gastrointestinal (n = 2)	8.5 ± 0.71	2.25 ± 2.48
Other (n = 2)	8.5 ± 0.71	4.75 ± 1.77
**Opioid analgesics (used alone and in combinations)** (n = 44)		
**Tramadol**		12 (27.27%)
**Codeine**		9 (20.45%)
**Morphine**		9 (20.45%)
**Fentanyl**		4 (9.09%)
**Pethidine**		2 (4.54%)
Methadone		1 (2.27%)
Oxycodone		1 (2.27%)
**Combined Opioid Usage**		
**Morphine and Fentanyl**		2 (4.54%)
**Fentanyl and Tramadol**		2 (4.54%)
**Morphine and Tramadol**		1 (2.27%)
**Codeine, Morphine, and Tramadol**		1 (2.27%)

**Table 3:** The mean and standard deviation (SD) of morphine equivalent doses (MEDs) for each prescribed opioid.

Average initial morphine equivalent dose before treatment (mg/d ± SD) (n = 44)	142 ± 157
**Single opioid**	
Tramadol (n = 12)	74.58 ± 79.47
Codeine (n = 9)	20.54 ± 22.71
Morphine (n = 9)	200 ± 211.82
Fentanyl (n = 4)	323.12 ± 151.67
Pethidine (n = 2)	100 ± 0
Methadone (n = 1)	70.5
Oxycodone (n = 1)	450
**Combined opioids**	
Morphine and Fentanyl (n = 2)	315 ± 75
Fentanyl and Tramadol (n = 2)	213.75 ± 116.25
Morphine and Tramadol (n = 1)	90
Codeine, Morphine, and Tramadol (n = 1)	41.6

## Data Availability

The original contributions presented in the study are included in the article material, further inquiries can be directed to the corresponding author.
